# Knowledge, attitudes, and practices related to Coronavirus disease 2019 during the outbreak among workers in China: A large cross-sectional study

**DOI:** 10.1371/journal.pntd.0008584

**Published:** 2020-09-17

**Authors:** Zhi-Hao Li, Xi-Ru Zhang, Wen-Fang Zhong, Wei-Qi Song, Zheng-He Wang, Qing Chen, Dan Liu, Qing-Mei Huang, Dong Shen, Pei-Liang Chen, Ang Mao, Duo Zhang, Xingfen Yang, Xian-Bo Wu, Chen Mao

**Affiliations:** 1 Department of Epidemiology, School of Public Health, Southern Medical University, Guangzhou, Guangdong, China; 2 Department of Epidemiology and Statistics, School of Public Health, Chengdu Medical College Chengdu, Sichuan, China; 3 Food Safety and Health Research Center, School of Public Health, Southern Medical University, Guangdong, China; WRAIR, UNITED STATES

## Abstract

Coronavirus disease 2019 (COVID-19) has recently emerged as a global threat. Understanding workers’ knowledge, attitudes, and practices (KAP) regarding this new infectious disease is crucial to preventing and controlling it. This study aimed to assess KAP regarding COVID-19 during the outbreak among workers in China. The present study was part of a cross-sectional online survey study conducted based on a large labor-intensive factory, which has 180,000 workers from various Chinese provinces, from 2 February 2020 to 7 February 2020. KAP related to COVID-19 were measured by 32 items, each item was measured with an agree/disagree/unclear format, and only correct responses were given 1 point. KAP regarding COVID-19 were measured with 20 items, 6 items and 6 items, respectively. A total of 123,768 valid responses (68.8%) were included in the analysis. Generally, the levels of knowledge (mean: 16.3 out of 20 points), attitudes (mean: 4.5 out of 6 points), and practices (mean: 5.8 out of 6 points) related to COVID-19 were high. Only 36,373 respondents (29.4%) disagreed that gargling with salt water is effective in protecting against COVID-19. Moreover, older respondents had decreased levels of knowledge and practices related to COVID-19 (both P values for the trend <0.001), while better-educated respondents had increased levels of knowledge and practices related to COVID-19 (both P values for the trend <0.001). These results suggest that Chinese workers are highly aware of COVID-19, but health authorities still need to provide correct information on COVID-19 prevention and strengthen health interventions, particularly for older and less-educated workers.

## Author summary

The Coronavirus disease 2019 (COVID-19) has recently emerged as a global threat. It is important to understanding workers’ knowledge, attitudes, and practices (KAP) related to COVID-19. In this study, we conducted a large cross-sectional online survey base on a large labor-intensive factory to determine the status of COVID-19-related KAP during the outbreak among workers in China. We found that Chinese workers had strong awareness of COVID-19 but also had some misconceptions. Moreover, this study indicated that lower levels of knowledge and practices related to COVID-19 among older and less-educated workers. The results suggested that health authorities still need to ensure correct information on COVID-19 prevention and strengthen health interventions, particularly for older and less-educated workers.

## Introduction

In December 2019, a cluster of cases of pneumonia caused by severe acute respiratory syndrome coronavirus 2 (SARS-CoV-2)[1], now called coronavirus disease 2019 (COVID-19)[2], was identified in Wuhan, Hubei Province, China[3, 4]. The disease has since spread rapidly from Wuhan to other cities (especially in Hubei Province) and caused more than 40,000 confirmed cases across 25 countries and 5 continents as of February 10, 2020[5]. To date, there are no vaccines or effective treatments for COVID-19[6], which is a major challenge and currently one of the most serious public health and social problems[7]. Understanding COVID-19 and taking effective preventive measures are important.

The importance of public education and community engagement in outbreak responses is well established[8–11]. Previous studies have indicated that surveys on knowledge, attitudes and practices (KAP) have helped inform many outbreak responses[12–15]. For example, Kobayashi and colleagues reported that Ebola-related KAP could be used to inform efforts to promote ongoing health awareness and messaging to address specific fears, misperceptions, and practices regarding Ebola[15]. However, regarding COVID-19, although enhanced surveillance and further investigation are ongoing[16], the vast majority of current studies have focused on COVID-19 etiology[16, 17], clinical characteristics[3, 18–21], sources or intermediate hosts of SARS-Cov-2[16, 22], and therapies or vaccines[23, 24]. No study has thus far focused on public KAP related to COVID-19, let alone COVID-19-related KAP among workers, who constitute the important group for prevention and control of the epidemic because of their large numbers and high crowd density.

Therefore, based on a sample of approximately 180,000 factory workers from a large factory, we conducted a large cross-sectional online survey to determine the status of COVID-19-related KAP during the outbreak among workers in China and to provide information to support the formulation of COVID-19 prevention and control strategies.

## Methods

### Ethics statement

This study was reviewed and approved by the Southern Medical University ethics committee, and written consent was obtained from all the respondents.

### Settings and study population

To describe the status of workers’ KAP related to COVID-19 during a large outbreak of COVID-19, we conducted a cross-sectional study based on a large labor-intensive factory in Shenzhen, China, which has 180,000 factory workers from various Chinese provinces. Data were collected online between 2 February 2020 and 7 February 2020 via the Chinese survey website Wenjuanxing (https://www.wjx.cn/), which is an online large-scale free questionnaire platform that has an innovative editing interface and a result analysis interface. The collected data include the following: sociodemographic characteristics, KAP and access to COVID-19-related information.

A total of 180,000 workers were invited to participate in the survey, and we received responses from 142,584 (79.2% response rate). While questionnaires completed by respondents with the same IP address, the second record was excluded (*n* = 15,035) since they were considered as an overlapping response. Moreover, questionnaires completed by respondents <510 seconds (*n* = 3781), because casually clicked to answer, were excluded from the analyses. In total, our analyses included data from 123,768 respondents (68.8% effective response rate) (S1 Fig).

### Survey questionnaire

The questions used to collect information were all closed ended. The sociodemographic characteristics included age (<25, 25–34, 35–44, and ≥45 years), sex, living area in the last two weeks (Hubei or other provinces), educational levels (college degree or above, high school, junior high school, and primary school and below), marital status (single, married, divorced, and others), and have confirmed cases in family members or relatives (yes, and no). Moreover, KAP related to COVID-19 were measured by 32 items, each item was measured via an agree/disagree/unclear format, and only correct responses were given 1 point. Knowledge of COVID-19 was based on a 20-item scale that assessed workers’ understanding of COVID-19 transmission, symptoms, and the differences between COVID-19 and other respiratory diseases. For evaluation, respondents who obtained scores >15 were considered to have “good” knowledge of COVID-19, and those who had scores ≤15 points were considered to have “poor” knowledge. Attitudes regarding COVID-19 were measured via a 6-item scale that assessed attitudes towards preventive measures, willingness, and concerns; respondents who answered ≤4 items correctly were considered to have “poor” understanding, whereas those who responded to >4 statements correctly were considered to have “good” understanding. Practices were measured on a 6-item scale that assessed personal hygiene practices and countermeasures. A score > 4 by an individual respondent was considered to indicate “good” practices. Two additional questions were asked: (i) Are you concerned about COVID-19-related information? (very concerned, concerned, not concerned); (ii) Are you feel panic about COVID-19? (a great deal of panic, panic, little panic, or none); (iii) How do you access information about COVID-19? (mobile social software [WeChat, QQ and Weibo], office website [government websites], TV, newspaper, and others).

### Statistical analysis

The categorical variables were described as numbers (%) and 95% confidence intervals (CIs). The continuous variables were presented using means and standard deviations (SD). Adjusted odds ratios (AOR) and their 95% CIs were calculated by multiple logistic regression as indicators of the strength of associations. All statistical analyses were performed using R version 3.6.2 (R Development Core Team, 2018); P<0.05 (two-sided) was considered statistically significant.

## Results

### Characteristics of the study population

Table 1 shows the characteristics of the study respondents. Among all 123,768 respondents, 36,438 (29.4%) respondents were women, the mean age was 30.3 years (SD: 6.4 years), and 38,499 (31.1%) respondents attained a college degree or above. A total of 9924 (8.0%) respondents had lived in Hubei Province for the last two weeks. Most (69,617; 56.2%) respondents were unmarried (Table 1). In addition, 27,045 (21.9%) respondents felt panic about COVID-19.

**Table 1:** Characteristics of workers in China, February 2-February 7, 2020 (*n* = 123,768).

Characteristic		Number (%)	95% CI
Sex			
	Men	87330 (70.6)	70.3–70.8
	Women	36438 (29.4)	29.2–29.7
Age group, years			
	<25	24476 (19.8)	19.6–20.0
	25–34	69847 (56.4)	56.2–56.7
	35–44	26352 (21.3)	21.1–21.5
	≥45	3093 (2.5)	2.4–2.6
Education levels			
	College degree or above	38499 (31.1)	30.8–31.4
	High school	48522 (39.2)	38.9–39.5
	Junior high school	35682 (28.8)	28.6–29.1
	Primary school and below	1065 (0.9)	0.8–0.9
Marital status			
	Unmarried	69617 (56.2)	56.0–56.5
	Married	49983 (40.4)	40.1–40.7
	Divorced	2788 (2.3)	2.2–2.3
	Others	1380 (1.1)	1.1–1.2
Living area in last two weeks			
	Hubei Province	9924 (8.0)	7.8–8.1
	Other provinces	113844 (92.0)	91.8–92.1
Family members or relatives infected			
	No	123636 (99.9)	99.9–99.9
	Yes	132 (0.1)	0.1–0.1

CI: confidence interval.

### Knowledge

The mean knowledge score was 16.3 points (SD =  3.1) out of a total possible score of 20 points. Most (14/20) knowledge questions had a high accuracy rate (> 85%). For example, 112,235 (90.7%) respondents agreed that the main symptoms of COVID-19 are fever, fatigue and dry cough, and most respondents agreed that washing hands (116,158; 93.9%) and avoiding going to crowded places (119,417; 96.5%) can prevent COVID-19 infection (Table 2). However, among all 123,768 respondents, only 40,327 (32.6%) disagreed that all patients with COVID-19 infection have fever symptoms, only 36,373 (29.4%) disagreed that gargling with salt water is effective in protecting against COVID-19, and only 58978 (47.7%) disagreed that vitamin C and Banlangen granules are effective for protecting against COVID-19.

**Table 2:** Questions, preferred responses, and participants’ responses from the knowledge, attitudes, and practices survey among workers in China, February 2-February 7, 2020.

Questions	Preferred response	Number (%)	95% CI
**Knowledge**			
The main symptoms of COVID-19 include fever, fatigue and dry cough	Agree	112235 (90.7)	90.5–90.8
Fever develops in all patients with COVID-19 infection	Disagree	40327 (32.6)	32.3–32.8
Patients with COVID-19 infection are not contagious if they do not have a fever	Disagree	94926 (76.7)	76.5–76.9
No specific antiviral has been approved for the treatment of COVID-19, but supportive care and symptomatic treatment can be highly effective for those infected	Agree	110393 (89.2)	89.0–89.4
Not all patients with COVID-19 infection will develop critical cases; rather, only cases of elderly people or those with underlying chronic diseases will be critical	Agree	75423 (60.9)	60.7–61.2
Children and infants don’t need to take preventive measures against COVID-19	Disagree	108776 (87.9)	87.7–88.1
Eating or having contact with wild animals can cause COVID-19 infections	Agree	114058 (92.2)	92.0–92.3
COVID-19 can spread through small droplets	Agree	118239 (95.5)	95.4–95.6
COVID-19 can spread through direct contact	Agree	114536 (92.5)	92.4–92.7
Wearing medical face masks is effective in protecting against COVID-19	Agree	82431 (66.6)	66.3–66.9
Living, studying or working together with a person who has been diagnosed with COVID-19 may lead to infection	Agree	118383 (95.6)	95.5–95.8
Living in the same ward as a person who has been diagnosed with COVID-19 may lead to infection	Agree	119703 (96.7)	96.6–96.8
Taking the same bus or train with a person who has been diagnosed with COVID-19 may lead to infection	Agree	118330 (95.6)	95.5–95.7
Avoiding going to crowded places can protect against COVID-19	Agree	119417 (96.5)	96.4–96.6
Frequent hand washing is effective in protecting against COVID-19	Agree	116158 (93.9)	93.7–94.0
Staying at home is effective in protecting against COVID-19	Agree	119703 (96.7)	96.6–96.8
Gargling with salt water is effective in protecting against COVID-19	Disagree	36373 (29.4)	29.1–29.6
Vitamin C and Banlangen granules are effective in protecting against COVID-19	Disagree	58978 (47.7)	47.4–47.9
Early isolation of and care for patients is effective in reducing the risk of transmission of COVID-19	Disagree	118017 (95.4)	95.2–95.5
People who are in close contact with someone with a confirmed case of COVID-19 infection should be isolated and observed as soon as possible, and the medical observation period is often 14 days	Agree	119299 (96.4)	96.3–96.5
**Attitudes**			
COVID-19 is a serious disease	Agree	114235 (92.3)	92.1–92.4
I am worried about being infected with COVID-19	Agree	63566 (51.4)	51.1–51.6
I am afraid of cured patients who were previously infected with COVID-19	Disagree	58213 (47.0)	46.7–47.3
I could tell if I had symptoms of COVID-19	Agree	87991 (77.1)	76.9–77.3
I know how to protect myself from COVID-19 infection	Agree	116446 (94.1)	94.0–94.2
If I show symptoms of COVID-19, I know where to go for treatment	Agree	114200 (92.3)	92.2–92.4
**Practices**			
I understand and follow the standards for wearing a mask during epidemics	Agree	120880 (97.7)	97.6–97.8
I understand and follow the standards for washing hands during epidemics	Agree	120928 (97.7)	97.6–97.8
I stay at home as much as possible except when necessary (such as for medical treatment and food purchases) during epidemics	Agree	121226 (97.9)	97.9–98.0
I do not trust or forward false and unverified information during epidemics	Agree	120701 (97.5)	97.4–97.6
I actively forward official information and take the initiative to share scientific information during epidemics	Agree	113360 (91.6)	91.4–91.8
If I show symptoms of COVID-19, I will actively seek treatment	Agree	120104 (97.0)	96.9–97.1

CI: confidence interval.

### Attitudes

The mean attitude score was 4.5 points (SD = 1.2) out of 6 points. Among all 123,768 respondents, 114,235 (92.3%) agreed that COVID-19 is a serious disease, and 114,200 (92.3%) agreed that if they showed symptoms of COVID-19, they would know where to seek treatment (Table 2). However, only 58,213 (47.0%) disagreed with the statement “I am afraid of cured patients who were previously infected with COVID-19”.

### Practices

The mean practices score was 5.8 points (SD = 0.8) out of 6 points. All practice questions had accuracy rates higher than 90% (range: 91.6%-97.9%) (Table 2). For example, among all 123,768 respondents, 120,880 (97.7%) agreed that they understood and followed the standards for wearing a mask during epidemics.

### Factors associated with knowledge, attitudes, and practices regarding COVID-19

Detailed information on univariate analysis of factors associated with poor knowledge, attitudes, and practices related to COVID-19 is shown in S1 Table. Multivariate analysis of COVID-19 knowledge shows that older respondents had less knowledge of COVID-19 (P for trend < 0.05; <25 years *versus* ≥45 years age groups, AOR: 1.16, 95% CI 1.07–1.27), while respondents with higher education levels had more knowledge of COVID-19 (P for trend < 0.05; primary school and below *versus* college degree or above, AOR: 0.19, 95% CI: 0.17–0.21) (Table 3). Respondents whose family or relatives was COVID-19 infected, had less of good knowledge (AOR: 3.82, 95% CI: 2.67–5.46) and good practices (AOR: 3.23, 95% CI: 1.76–5.92) related to COVID-19. Similarly, in multivariate analysis of practices related to COVID-19, older respondents scored lower on practices related to COVID-19 (P for trend < 0.05), while respondents with higher education levels scored higher (P for trend < 0.05) (Table 3).

**Table 3:** Multivariate analysis of factors associated with poor knowledge, attitudes, and practices related to COVID-19 among workers in China, February 2-February 7, 2020.

Characteristic	Knowledge		AOR (95% CI)	*P* value	Attitudes		AOR (95% CI)	*P* value	Practices		AOR (95% CI)	*P* value
	Good	Poor			Good	Poor			Good	Poor		
Sex												
Women	26709 (73.3)	9729 (26.7)	1.00 (ref.)	-	21665 (59.5)	14773 (40.5)	1.00 (ref.)	-	35396 (97.1)	1042 (2.9)	1.00 (ref.)	-
Men	63905 (73.2)	23425 (26.8)	0.99 (0.47–0.61)	0.543	52001 (59.5)	35329 (40.5)	1.00 (0.98–1.03)	0.901	84824 (97.1)	2506 (2.9)	1.00 (0.93–1.08)	0.982
Age group, y												
<25	18464 (75.4)	6012 (24.6)	1.00 (ref.)	-^**#**^	14510 (59.3)	9966 (40.7)	1.00 (ref.)	-	23901 (97.7)	575 (2.3)	1.00 (ref.)	-^**#**^
25–34	51021 (73.0)	18826 (27.0)	1.08 (1.04–1.12)	<0.001	41596 (59.6)	28251 (40.4)	1.00 (0.97–1.03)	0.958	67794 (97.1)	2053 (2.9)	1.16 (1.05–1.27)	0.002
35–44	18924 (71.8)	7428 (28.2)	1.13 (1.09–1.18)	<0.001	15682 (59.5)	10670 (40.5)	1.01 (0.98–1.05)	0.584	25542 (96.9)	810 (3.1)	1.16 (1.04–1.29)	0.010
≥45	2205 (71.3)	888 (28.7)	1.16 (1.07–1.27)	<0.001	1878 (60.7)	1215 (39.3)	0.97 (0.90–1.05)	0.466	2983 (96.4)	110 (3.6)	1.31 (1.07–1.62)	0.010
Education level												
Primary school and below	529 (49.0)	536 (50.3)	1.00 (ref.)	-^**#**^	556 (52.2)	509 (47.8)	1.00 (ref.)	-	938 (88.1)	127 (11.9)	1.00 (ref.)	-^**#**^
Junior high school	22473 (63.0)	13209 (37.0)	0.59 (0.52–0.67)	<0.001	19390 (54.3)	16292 (45.7)	0.93 (0.82–1.05)	0.232	34208 (95.9)	1474 (4.1)	0.33 (0.27–0.40)	<0.001
High school	35065 (72.3)	13457 (27.7)	0.39 (0.34–0.44)	<0.001	28797 (59.3)	19725 (40.7)	0.76 (0.67–0.86)	<0.001	47343 (97.6)	1179 (2.4)	0.20 (0.16–0.24)	<0.001
College degree or above	32547 (84.5)	5952 (15.5)	0.19 (0.17–0.21)	<0.001	24923 (64.7)	13576 (35.3)	0.60 (0.53–0.68)	<0.001	37731 (98.0)	768 (2.0)	0.17 (0.14–0.20)	<0.001
Married status												
Married	28941 (77.9)	11042 (22.1)	1.00 (ref.)	-	29509 (59.0)	20474 (41.0)	1.00 (ref.)	-	49141 (98.3)	842 (1.7)	1.00 (ref.)	-
Single	48779 (70.1)	20838 (29.9)	1.43 (1.39–1.46)	<0.001	41873 (60.1)	27744 (39.9)	0.94 (0.91–0.96)	<0.001	67093 (96.4)	2524 (3.6)	2.08 (1.92–2.25)	<0.001
Divorced	2055 (73.7)	733 (26.3)	1.07 (0.98–1.17)	0.151	1609 (57.7)	1179 (42.3)	0.99 (0.92–1.07)	0.798	2717 (97.5)	71 (2.5)	1.36 (1.06–1.74)	0.014
Others	839 (60.8)	541 (39.2)	1.82 (1.62–2.03)	<0.001	675 (48.9)	705 (51.1)	1.38 (1.24–1.54)	<0.001	1269 (92.0)	111 (8.0)	4.16 (3.38–5.13)	<0.001
Living area in last two weeks												
Hubei province	8067 (81.3)	1857 (18.7)	1.00 (ref.)	-	6039 (60.9)	3885 (39.1)	1.00 (ref.)	-	9750 (98.2)	174 (1.8)	1.00 (ref.)	-
Other provinces	82547 (72.5)	31297 (27.5)	1.43 (1.35–1.50)	<0.001	67627 (59.4)	46217 (40.6)	1.02 (0.98–1.07)	0.311	110470 (97.0)	3374 (3.0)	1.43 (1.22–1.67)	<0.001
Family members or relatives infected												
No	90557 (73.2)	33079 (26.8)	1.00 (ref.)	-	73588 (59.5)	50048 (40.5)	1.00 (ref.)	-	120100 (97.1)	3536 (2.9)	1.00 (ref.)	-
Yes	57 (43.2)	75 (56.8)	3.82 (2.67–5.46)	<0.001	78 (59.1)	54 (40.9)	1.01 (0.71–1.44)	0.943	120 (90.9)	12 (9.1)	3.23 (1.76–5.92)	<0.001

AOR: Adjusted odds ratios; CI: confidence interval.

^**#**^ P for trend <0.05.

### Sources of receiving information

**Fig 1:**
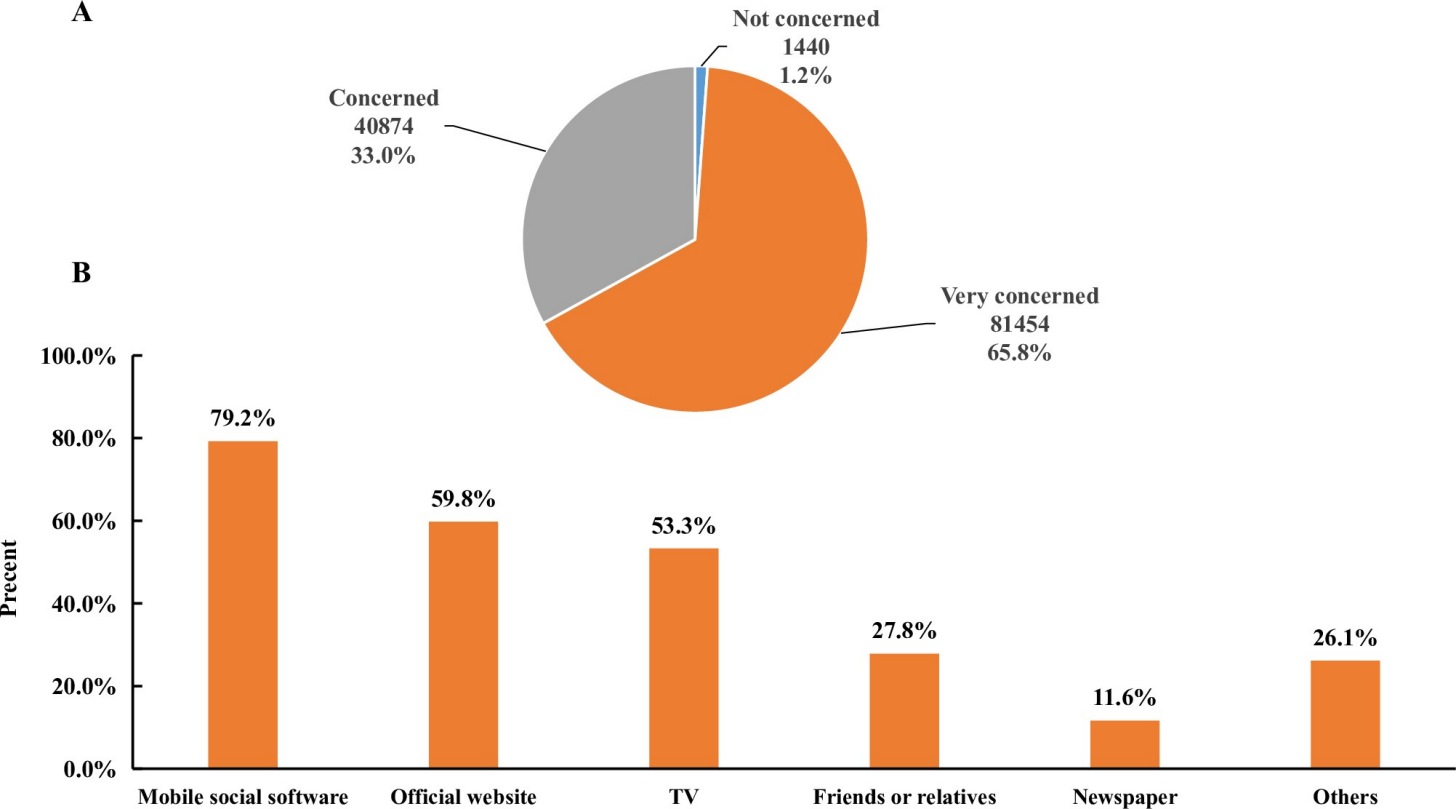
Attention (A) and access (B) to COVID-19-related information.

Of all 123,768 respondents, most (81,454 [65.8%] were very concerned and 40,874 [33.0%] were concerned) were concerned about COVID-19-related information. In addition, the three main ways for respondents to access COVID-19-related information were mobile social software (WeChat, QQ and Weibo, 79.2%), office websites (government websites, 59.8%), and TV (53.3%) (Fig 1).

## Discussion

### Principal findings

Our study, involving over 120,000 respondents, conducted a rapid assessment of workers’ KAP related to COVID-19 during a large outbreak and found that levels of knowledge (mean: 16.3 out of 20 points), attitudes (mean: 4.5 out of 6 points), and practices (mean: 5.8 out of 6 points) of COVID-19 were relatively high. However, gaps in knowledge, misconceptions and discriminatory attitudes regarding COVID-19 were common, for example, only 29.4% respondents disagreed that gargling with salt water is effective in protecting against COVID-19, and only 47.7% respondents disagreed that vitamin C and Banlangen granules are effective for protecting against COVID-19. Moreover, older respondents had lower levels of knowledge and practices related to COVID-19 (both P for trend < 0.05), while better-educated respondents had higher levels of knowledge and practices related to COVID-19 (both P for trend < 0.05).

In contrast to previous studies of KAP related to other infectious diseases (Ebola, H7N9, etc.)[25–27], our study showed that COVID-19 awareness was high among workers in China, although COVID-19 has not been spreading for a long time (nearly two months since the outbreak). The most important reason is related to the measures taken by China, including extending the Spring Festival holiday, postponing the start of schools and factories, and adopting transport restrictions in various areas; blocking Wuhan's trains, planes and other traffic[28]; and propagating knowledge of COVID-19 through various media and official guidelines[29]. In addition, because of the strong publicity across the country and communities at all levels and the experience of battling SARS, the vast majority of Chinese people are willing to pay attention to the epidemic through various channels, obtain correct knowledge, hold positive attitudes, and take necessary precautions.

Although most people try to obtain correct information about COVID-19 through various media, most workers lack professional knowledge and still believe some rumors. For example, only 36,373 (29.4%) of respondents disagreed that gargling with salt water is effective in protecting against COVID-19, and only 58,978 (47.7%) disagreed that vitamin C and Banlangen granules are effective in protecting against COVID-19. Additionally, although the early recognition of symptoms is key to early healthcare-seeking behavior, only 32.6% of respondents disagreed that all patients with COVID-19 infection had fever symptoms. False or misleading information is dangerous, as it can cause widespread public reluctance to adopt well-founded infection control measures promoted by health authorities and thus delay essential interventions[30]. Combating the spread of rumors and misinformation is very important for epidemic prevention and control[30]; therefore, health authorities in China should carry out targeted public information campaigns to promote the spread of accurate knowledge about COVID-19 symptoms and transmission modes and methods to protect oneself against COVID-19. In addition, fear of COVID-19 and of cured patients who were previously infected with COVID-19 was prevalent among workers, possibly because of the highly infectious nature of SARS-CoV-2, lack of effective treatment or personal protective equipment and low food stocks at home due to travel restrictions. Targeted educational messages and assurance of the provision of protective equipment and food supplies might help to alleviate some of these fears and give the public a sense of security and confidence. Furthermore, health authorities need to identify and overcome barriers to access to health care to ensure the early diagnosis and treatment of COVID-19 patients. Moreover, our results show that most workers were concerned about COVID-19-related information and got the information through mobile phone software, indicating that new media, especially mobile phone software, have played an important role in quickly disseminating COVID-19-related messages. Therefore, health authorities may prioritize the use of new media to provide the public with knowledge and methods that can reduce or eliminate the infection or harm and guide the public to take effective protective measures when public health emergencies occur.

Similar to the findings of previous studies[15, 31, 32], for the knowledge and practices related to COVID-19, better-educated individuals had higher scores, which were partially explained by the fact that better-educated workers could process information more quickly, and may be more capable of distinguishing correct information and acting upon it. Moreover, older respondents had less knowledge and practices related to COVID-19 than younger respondents. Health authorities should, therefore, strengthen public information and education for older and less-educated workers to increase their awareness of COVID-19 and improve practices.

### Limitations

Several potential limitations of this study should be considered. First, given the limited resources available and the time-sensitive nature of this emergency, it was not feasible for the sample to include individuals from all Chinese provinces. However, the more than 120,000 valid responses covered most of the provinces in China, thus decreasing the likelihood of sampling bias. Second, self-reported behaviors may not always be aligned with actual practices: respondents may have provided socially desirable responses[33], especially due to the high awareness of COVID-19 and widespread sensitization and education efforts at the time. Third, because the survey was conducted online in Wenjuanxing, respondents may not have provided completely independent answers, as they lived together. Finally, a standardized form was used for the survey, but none of the responses was open ended. Therefore, limited information was available beyond the three options of “agree”, “disagree” and “unclear”.

Notwithstanding these limitations, this KAP study is believed to be the first survey conducted during this outbreak to assess the effectiveness of COVID-19-related messaging among workers in China. Furthermore, this KAP study was conducted during the COVID-19 outbreak; hence, the findings directly informed the development of a national social mobilization strategy and provided a baseline for evaluating COVID-19 prevention, control and care efforts among workers throughout the remainder of the epidemic.

### Conclusions

The results of this large-scale KAP survey showed that Chinese workers had strong awareness of COVID-19 but also had some knowledge misconceptions. Moreover, we found lower levels of knowledge and practices related to COVID-19 among older and less-educated workers. These results suggested that health authorities need to ensure correct information on COVID-19 prevention and strengthen health interventions, particularly for older and less-educated workers, to combat rumors and misinformation and reduce public panic.

## Supporting information

S1 Table Univariate analysis of factors associated with poor knowledge, attitudes, and practices related to COVID-19 among workers in China, February 2-February 7, 2020. (DOC)

S1 Fig Flowchart for the selection of the analyzed study sample. (TIF)

S1 Data All relevant data. (XLSX)
