# Supplementary material for: Knowledge, attitudes, and practices related to Coronavirus disease 2019 during the outbreak among workers in China: A large cross-sectional study
Source: PLoS Negl Trop Dis. 2020 Sep 17;14(9):e0008584. doi: 10.1371/journal.pntd.0008584 (PMC7498029; doi:10.1371/journal.pntd.0008584)
Supplement: S1 Table — (DOC) [file pntd.0008584.s001.doc]

**S1 Table. Univariate analysis of factors associated with poor knowledge, attitudes, and practices related to COVID-19 among workers in China, February 2-February 7, 2020.**

| **Characteristic** | **Knowledge** | | ***P* value** | **Attitudes** | | ***P* value** | **Practices** | | ***P* value** |
| --- | --- | --- | --- | --- | --- | --- | --- | --- | --- |
|  | **Good** | **Poor** |  | **Good** | **Poor** |  | **Good** | **Poor** |  |
| Sex |  |  | 0.660 |  |  | 0.778 |  |  | 0.939 |
| Women | 26709  (73.3) | 9729  (26.7) |  | 21665  (59.5) | 14773  (40.5) |  | 35396  (97.1) | 1042  (2.9) |  |
| Men | 63905  (73.2) | 23425  (26.8) |  | 52001  (59.5) | 35329  (40.5) |  | 84824  (97.1) | 2506  (2.9) |  |
| Age group, y |  |  | <0.001 |  |  | 0.485 |  |  | <0.001 |
| <25 | 18464  (75.4) | 6012  (24.6) |  | 14510  (59.3) | 9966  (40.7) |  | 23901  (97.7) | 575  (2.3) |  |
| 25-34 | 51021  (73.0) | 18826  (27.0) |  | 41596  (59.6) | 28251  (40.4) |  | 67794  (97.1) | 2053  (2.9) |  |
| 35-44 | 18924  (71.8) | 7428  (28.2) |  | 15682  (59.5) | 10670  (40.5) |  | 25542  (96.9) | 810  (3.1) |  |
| ≥45 | 2205  (71.3) | 888  (28.7) |  | 1878  (60.7) | 1215  (39.3) |  | 2983  (96.4) | 110  (3.6) |  |
| Education level |  |  | <0.001 |  |  | <0.001 |  |  | <0.001 |
| Primary school and below | 529  (49.0) | 536  (50.3) |  | 556  (52.2) | 509  (47.8) |  | 938  (88.1) | 127  (11.9) |  |
| Junior high school | 22473  (63.0) | 13209  (37.0) |  | 19390  (54.3) | 16292  (45.7) |  | 34208  (95.9) | 1474  (4.1) |  |
| High school | 35065  (72.3) | 13457  (27.7) |  | 28797  (59.3) | 19725  (40.7) |  | 47343  (97.6) | 1179  (2.4) |  |
| College degree or above | 32547  (84.5) | 5952  (15.5) |  | 24923  (64.7) | 13576  (35.3) |  | 37731  (98.0) | 768  (2.0) |  |
| Marital status |  |  | <0.001 |  |  | <0.001 |  |  | <0.001 |
| Married | 28941  (77.9) | 11042  (22.1) |  | 29509  (59.0) | 20474  (41.0) |  | 49141  (98.3) | 842  (1.7) |  |
| Single | 48779  (70.1) | 20838  (29.9) |  | 41873  (60.1) | 27744  (39.9) |  | 67093  (96.4) | 2524  (3.6) |  |
| Divorced | 2055  (73.7) | 733  (26.3) |  | 1609  (57.7) | 1179  (42.3) |  | 2717  (97.5) | 71  (2.5) |  |
| Others | 839  (60.8) | 541  (39.2) |  | 675  (48.9) | 705  (51.1) |  | 1269  (92.0) | 111  (8.0) |  |
| Living area in last two weeks |  |  | <0.001 |  |  | 0.005 |  |  | <0.001 |
| Hubei Province | 8067  (81.3) | 1857  (18.7) |  | 6039  (60.9) | 3885  (39.1) |  | 9750  (98.2) | 174  (1.8) |  |
| Other provinces | 82547  (72.5) | 31297  (27.5) |  | 67627  (59.4) | 46217  (40.6) |  | 110470  (97.0) | 3374  (3.0) |  |
| Family members or relatives infected |  |  | <0.001 |  |  | 0.920 |  |  | <0.001 |
| No | 90557  (73.2) | 33079  (26.8) |  | 73588  (59.5) | 50048  (40.5) |  | 120100  (97.1) | 3536  (2.9) |  |
| Yes | 57  (43.2) | 75  (56.8) |  | 78  (59.1) | 54  (40.9) |  | 120  (90.9) | 12  (9.1) |  |
