# Supplementary figures and images for: Knowledge, attitudes, and practices related to Coronavirus disease 2019 during the outbreak among workers in China: A large cross-sectional study
Source: PLoS Negl Trop Dis. 2020 Sep 17;14(9):e0008584. doi: 10.1371/journal.pntd.0008584 (PMC7498029; doi:10.1371/journal.pntd.0008584)

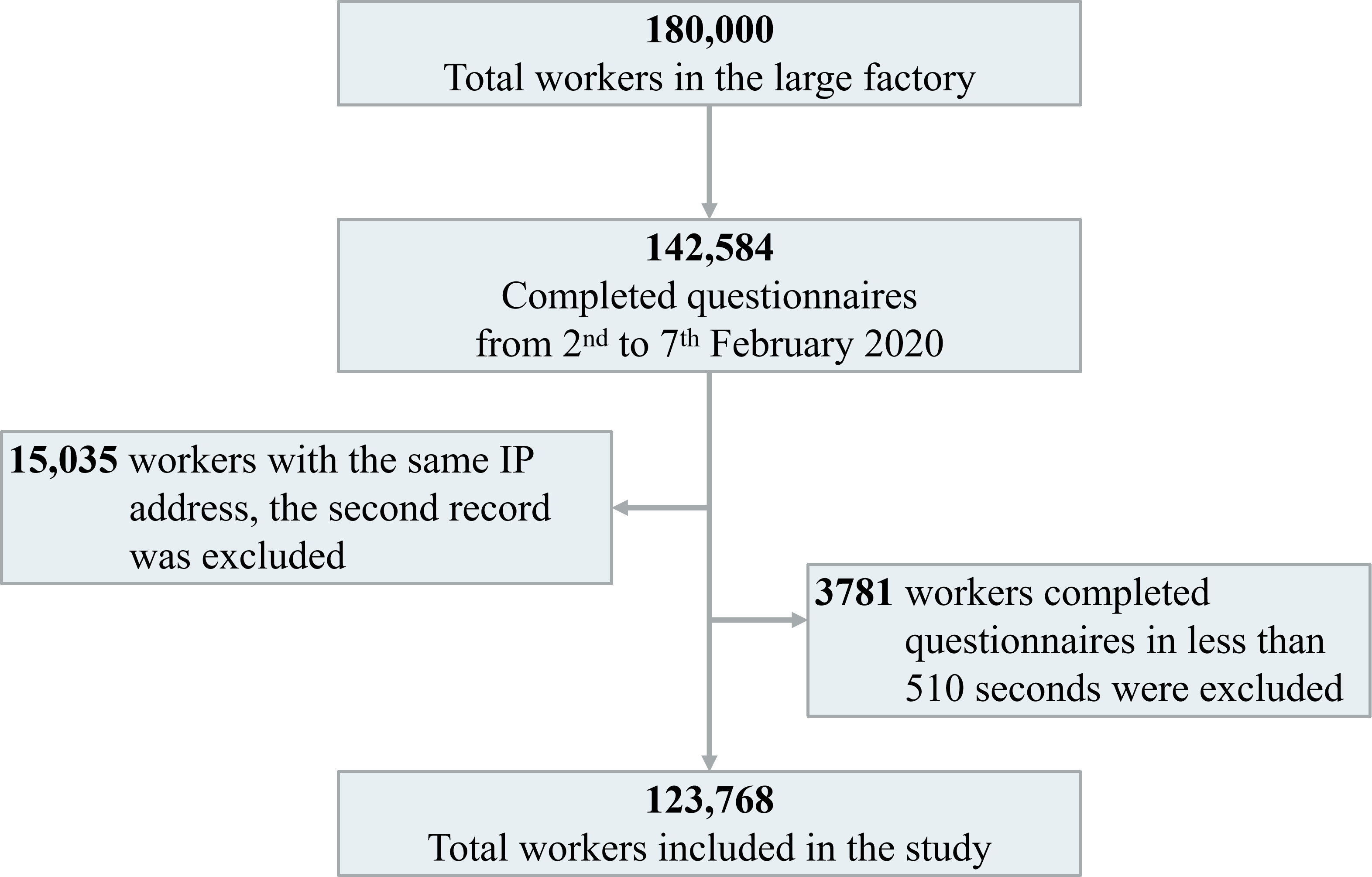

Supplement: S1 Fig — (TIF) [file pntd.0008584.s002.tif]
